# MoodJumper: An Exploration of Game Interface Preferences in Users With/Out Mood Disorder

**DOI:** 10.3389/fpubh.2019.00220

**Published:** 2019-08-08

**Authors:** Nilufar Baghaei, Sylvia Hach, Hai-Ning Liang, Marvin Brucker

**Affiliations:** ^1^Otago Polytechnic Auckland (OPAIC), Auckland, New Zealand; ^2^School of Community Studies, Unitec Institute of Technology, Auckland, New Zealand; ^3^Department of Computer Science and Software Engineering, Xi'an Jiaotong-Liverpool University, Suzhou, China

**Keywords:** game design, mood disorder, user preferences, user study, user interface design

## Abstract

Mental health conditions pose a major challenge to healthcare providers and society at large. The World Health Organization predicts that by 2030 mental illnesses will be the leading disease burden globally. Mental health services are struggling to meet the needs of users and arguably fail to reach large proportions of those in need. According to New Zealand Mental Health Foundation, one in five will experience a serious mood disorder, including depression, at some time in their life. Games for Health including those supporting mental health have recently gained a lot of attention. However, game interface preferences for users with a history of mental health conditions have not been systematically studied, making it difficult to determine what game features may attract and further engage users affected by mental health conditions. We present MoodJumper, a prototype Android mobile game, which enables players to jump to the top of the level by steering the avatar from platform to platform, gradually gaining height and collecting coins on the way up. We conducted a preliminary study (*n* = *25*), in which participants were able to modify different settings of the game (background color, dark/light, character movement, gender, and music), while their gaming behavior was tracked. The results show that regardless of self-reported history of mood disorder, the majority of participants prefer the dark and colored layout setting and there were no differences in gaming variables including session duration and high scores. This represents a first indication that history of mood disorder does not affect user preferences for game interface settings. It will be important to follow up with data on users currently affected by low mood. Systematic study of game interface preferences in users with mood disorder constitutes a vital step in being able to harness the potential power of games for supporting mental health.

## Introduction

Mental health conditions pose a major challenge to healthcare providers and society at large. The World Health Organization predicts that by 2030, mental illnesses will be the leading disease burden globally (1). Mental health services are struggling to meet the needs of users and arguably fail to reach large proportions of those in need. There is growing financial pressures on mental health services (2, 3). According to Mental Health Foundation New Zealand, one in five will have a serious mood disorder, including depression, at some time in their life (3).

### Games for Health

Video games as a platform, since their beginnings in the 1970s, have become a widely distributed and relevant media phenomenon within most modern societies, impacting the daily lives of children and adolescents as well as adults. Due to their unique level of interactivity among other forms of media consumption, videogames have changed and expanded the way we experience and explore medial presentations. For this reason, scientific studies in the domain of games are plenty and diverse (4).

When talking about games, it is important to mention that the mobile market has seen an even more explosive growth (5) and as a consequence makes the medium of video games accessible for more people than ever before.

Early intervention can have significant positive impact on a person's prognosis, particularly important in affecting outcomes for young people (2, 6–9). Games for Health, including those supporting mental health, have recently attracted a lot of attention (10). Examples include games that provide Computerized Cognitive Behavior Therapy (CCBT) for people with depression and bipolar disorder (11–13).

### Game Interface Preferences

Computer and mobile games have proliferated in recent years. However, to the best of our knowledge, user interface preferences have not been studied in individuals with a history of mood disorder. Yet, it is well-known that mood can implicitly and explicitly affect cognition and behavior in a broad manner.

A good example of this is the mood congruency effect in memory. Briefly, mood congruency in memory describes the phenomenon of improved memory for information that is of a valence consistent with the mood of the individual (14). That is, when in a sad mood while memorizing unpleasant and pleasant words, unpleasant words are more easily recalled at a later stage (15).

Similar effects may be present with regard to preference for settings that are usually available as part of games. For example, a link between color and emotion has long been proposed (16). There is some empirical evidence that color preferences change with the mood of individuals, particularly for younger people (17, 18).

Similarly, music preference has been seen as an indicator of emotional vulnerability (19) and music selection for specific scenarios has, among other factors, been shown to be affected by emotion regulation style (20). Moreover, recent work in the recommender systems domain has recognized the importance of user affect and has demonstrated superior user acceptance when including user emotions in its algorithms (21).

A final game setting that may be of importance to study systematically regarding user preference is that of movement direction. The space-valence metaphor describes a relationship between vertical space and mood [e.g., (22)]. Specifically, upper visual space and upward body movement is typically associated with positive emotions and mood, while lower visual space and body movement toward the ground is associated with negative emotions and mood [e.g., (23, 24)].

User preference such as choosing color, music, and movement direction is influenced significantly by mood and is important to consider in game design and specifically the design of games for mental health for two reasons:

1. The availability of preferred settings may increase the uptake of the game. This is particularly important to consider when designing games for health, as compliance with pharmacological intervention (25), psychotherapy (26), and e-therapy (27) is typically associated with superior outcomes.
2. User preference can influence the effect a specific setting has on the user. Attention and engagement may be affected if a setting such as music is not consistent with the mood of the user [e.g., (20)].

The aim of our project was to design and develop a mobile game that allows users to switch between different settings, in order to evaluate putative differences in preferences for UI setting between players with and without a history of mood disorder. Our research question was whether users with self-reported mood disorder show different game interface preferences. The rest of the paper is organized as follows. Section Moodjumper Design describes the design of the MoodJumper game. The experiment and preliminary findings are discussed in section Experimental Setup and Preliminary Results. Section Conclusion & Future Work concludes the paper and outlines future plans.

## Moodjumper Design

**Figure 1:**
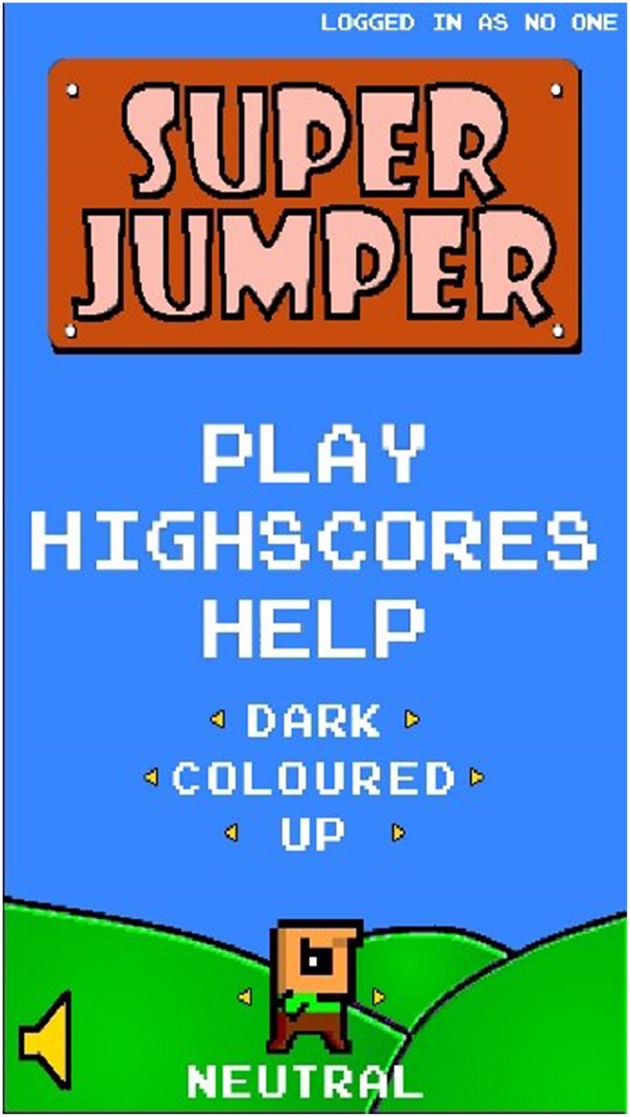
Menu screen of MoodJumper.

The MoodJumper game is developed for Android mobiles. We took an open source game and modified it in order to allow players to change the game design and set it to what they prefer. MoodJumper consists of two main screens, the menu screen and the play screen. The menu screen (see Figure 1) is the entry point for each participant. It contains clickable menu items to start the game (Play), to see high scores (High scores) and to click through a series of screen-shots that give tips on how to play the game (Help). Underneath, the participant (or a user/player) has the chance to click through and choose different settings and features within the game. The participant can choose between a dark and bright layout, between a colored and a black and white layout and between three different avatars, a male, female and neutral avatar. Furthermore, the participant can decide to either play with or without sounds (which include a looping soundtrack and sound effects within the game) as well as in which direction the avatar will jump (upwards or downwards).

**Figure 2:**
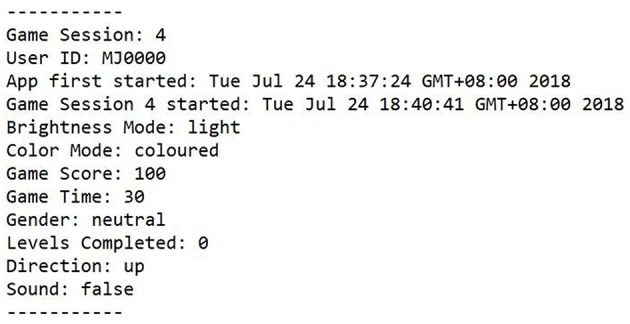
Example data of one game session.

Once the participant presses play and starts the game, a new game session is started and the participant's setting and feature choices, the time played in this session, the highscores and the user ID are saved in a text file, see Figure 2 for an example data set.

The goal of the game is to jump to the top of the level by steering the avatar from platform to platform, gradually gaining height and collecting coins on the way up. The jumping is done automatically and cannot be stopped by the participant, who can only give the avatar a direction to the left or right by tilting the tablet device we use for the experiment. With an increasing height, the participant will face flying enemies that move from side to side and, once it is touched by the avatar, that will end the game immediately for the participant. In the first level, the avatar can use the same platform three times before it destroys itself. In the second level the platform only allows two jumps on the same platform and level three only one jump per platform.

**Figure 3:**
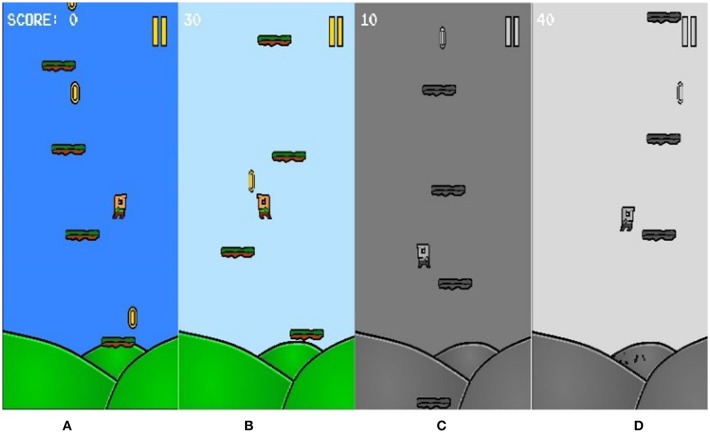
Example of various layout settings. **(A)** Dark/Colored **(B)** Bright/Colored **(C)** Dark/Black & White **(D)** Bright/Black & White.

The game session is over if the avatar touches one of the flying enemies or touches the bottom of the level (except for the bottom at the start of each level). Once a game session is over the participant is redirected to the menu screen again where the setting and feature choices can be altered and a new session can be started (see Figure 3 for different setting example).

## Experimental Setup And Preliminary Results

We carried out a pilot study with 25 participants (19 male, 6 female) in July 2018. The studies involving human participants were reviewed and approved the Research Ethics Sub-Committee at Xi'an Jiaotong-Liverpool University. Participation in the study was entirely voluntary. The experiment started with the participant being introduced to the purpose of this study and being asked to sign a standard consent form. The participants provided their written informed consent to take part in this study. After collecting the participant's demographic details (including age, gender, ethnicity, and familiarity with games), participants were asked about any history of mood disorder and invited to play the game for a duration of at least 20 min. Following the 20 min playtime (maximum 30 min), a last questionnaire was completed by each participant. This questionnaire asked about the overall enjoyment of playing the game as well as the preferred settings and features.

All participants were students at the ≪name removed for anonymity≫ University between the age of 19–30 years (M = 23). The majority (72%) of participants identified as Chinese while the rest identified with another ethnicity (African, Latin American, South-East Asian). Participants showed diverse videogame patterns. A total of 32% stated that they play occasionally, once every few months. Only 12% stated that they play at least every month, while 24% answered that they play every week and another 32% indicating that they play every day.

The single highest score obtained by each participant as well as the number of played game sessions were collected. The lowest high score obtained by a participant was 110, while the highest high score was 720 (M = 270). Participants played between 19 and 42 game sessions (M = 27) in their game time (minimum of 20 min playtime).

With regard to the preferred UI settings and features, the following summary shows the results of the post-experiment survey. A total of 60% of participants preferred the dark colored layout (dark and color setting activated) and 28% indicated preference for the light colored layout (light and color setting activated). While 8% preferred the dark black and white screen layout, only 4% preferred the combination of light and black and white settings activated. The preferences regarding the sound were similarly balanced, with 60% preferring to play with the sound on and 40% indicating they preferred playing without the sound. A clearer preference was observed when asking for the game direction feature. The majority of participants (96%) preferred the upward moving game direction, which means the avatar jumped from the bottom of the screen in an upward direction. Only 4% of participants (*n* = 1), preferred the avatar to jump from the top of the screen in a downward direction.

Consistent with data of a high prevalence of mood disorders in the student population worldwide, 64% of participants indicated a history of some form of mood disorder. For the following part of the results section, all participants who answered with yes in terms of history of mood disorder, are represented as “Group 1,” while “Group 2” contains participants not reporting a history of mood disorder. Collected results from participants of both groups are displayed in Table 1 for comparison.

**Table 1:** Comparison between Group 1 (participants with a self-reported history of mood disorder) and Group 2 (participants without a history of mood disorder).

**Group**	**Group 1**	**Group 2**
Highscore Range	110–720	160–440
Average Highscore	351.88	263.33
Session Range	13–42	17–30
Average Session	28.19	25.78
Preferences		
Dark Colored	62.5%	55.55%
Light Colored	25%	33.33%
Dark Black & White	12.5%	0%
Light Black & White	0%	11.11%
Moving Up	93.75%	100%
Moving Down	6.25%	0%
Music On	56.25%	66.67%
Music Off	43.75%	33.33%

Due to the higher number of participants in Group 1, the range of high scores achieved and the range of sessions being played was greater than in Group 2. There was no statistically significant difference between the groups with regard to the single highest score throughout the game time; Group 1 (351.88 ± 202.03), Group 2 (263.33 ± 101.73) *p* = 0.159. However, these results could be affected by the rather limited number of participants. When applying the same approach for the played sessions, taking the total number of played sessions per participant and comparing them between the two groups, no significant difference between Group 1 (28.19 ± 8.46) and Group 2 (25.78 ± 4.89) *p* = 0.376 was found. In terms of preferred settings and features, the tendencies are very similar for both groups. The majority of participants of both groups preferred the Dark and Colored layout setting (62.5% for Group 1 and 55.55% for Group 2), followed by the Light and Colored one (preferred by 25% in Group 1 and 33.33% in Group 2). Both groups have a small percentage of participants preferring either one of the Black and White layout style (one participant in Group 2 and two participants in Group 1). The same preference tendency can be observed for the feature of either moving the avatar upward or downward. Overall, only one participant, from Group 1, preferred the avatar moving down which results in a 100% preference for moving up in Group 2, compared to 93.75% in Group 1. While again the tendency is the same regarding the music settings in the game, showing that both groups prefer playing the game with enabled sounds, Group 1 shows slightly more balanced preferences (56.25% music on, 43.75% music off compared to 66.67% music on and 33.33% music off for Group 2).

Table 2 shows similar results, but this time comparing male and female participants overall (note: a row was added to show the distribution of participants with history of mood disorder among the gender groups). Similar to the previous group comparison, the raw data indicates that male and female participants show the same tendencies with only slight differences in the distribution. In general, male participants play less game sessions (average 24.68 compared to 35.67 for females) but score two times higher on average. The comparison was statistically comparable.

**Table 2:** Comparison between Male and Female participants.

**Group**	**Male**	**Female**
History of mood disorder (Yes)	63.16%	66.67%
Highscore Range	120–720	110–280
Average Highscore	365.26	176.67
Session Range	13–37	27–42
Average Session	24.68	35.67
Preferences		
Dark Colored	57.89%	66.67%
Light Colored	26.32%	33.33%
Dark Black and White	10.53%	0%
Light Black & White	5.26%	0%
Moving Up	100%	83.33%
Moving Down	0%	16.67%
Music On	57.89%	66.67%
Music Off	42.11%	33.33%

In the post-experiment survey, we asked participants to provide any comments they had about the game in general, if they enjoyed playing it and whether they had any suggestions for further improvement. Seven participants mentioned that the enemies within the game are too hard to avoid and/or that it should be possible to attack them. Four participants stated that the choice of character did not make any difference for them and that the neutral character and the male character are too similar to each other. Three participants expressed their dislike about the music and visual style of the game. In terms of game logic, three participants were unhappy with the way scores were earned and they felt it would be more motivational, if not only collected coins would give points but also getting higher with the character, as an additional reward. A few participants stated that they would have enjoyed playing the game more, if it was possible for them to change the sensitivity of the character reacting to the tilting of the tablet.

## Conclusion And Future Work

In this paper, we presented MoodJumper, an Android mobile game that allows players to change specific game settings to their preferred settings, therefore enabling the systematic study of user preference and the impact of congruency between a setting and user preference on gaming performance. Preference for user settings have not previously been examined in detail and, to our knowledge, preferences of users with a history of mood disorders have not been studied at all. Yet, with increasing demands on mental health services and games for health being developed in order to partially address these demands, investigations of user preferences are much needed. Based on previous literature showing that mood affects preferences for specific colors, music, and vertical movement direction, user preferences for these settings were studied here in a group of users with and without a history of self-reported mood disorder.

The preliminary results show that the majority of participants of both groups (with or without a self-reported history of mood disorder) prefer the dark and colored layout setting. The majority of participants in both groups wanted the character to move upwards and preferred the music on. We did not observe any statistically significant differences between the results of female and male participants. This represents a first indication that history of mood disorder does not affect user preferences for game interface settings. More studies are needed to examine the game interface preferences of users affected by mood disorder at the time of engaging with the game.

Future plans include improvements to the music and visual appeal of the characters, personalization options of MoodJumper based on user preference, gaming habits, frequency of play and more in-depth examination of players' engagement with the game. Further, a larger evaluation study with general population as well as people who have been diagnosed with mood disorder (as opposed to self-reported history) will be conducted. The systematic study of game interface preferences in users with mood disorder constitutes a vital step in being able to harness the potential power of games for supporting mental health. We believe our research paves the way for the systematic design of full-fledged therapeutic games for maximizing the engagement of people with mood disorder.

## Data Availability

The datasets generated for this study are available on request to the corresponding author.

## Author Contributions

All authors have had scientific contribution into this work (including design, implementation, evaluation, and write up phases), especially the first two authors, who had the most amount of contribution.

### Conflict of Interest Statement

The authors declare that the research was conducted in the absence of any commercial or financial relationships that could be construed as a potential conflict of interest. The handling editor declared a shared affiliation, though no other collaboration, with one of the authors NB at time of review.
